# Determinants of complementary feeding practices among children aged 6–24 months in urban slums of Pune, Maharashtra, in India

**DOI:** 10.1186/s41043-022-00342-6

**Published:** 2023-01-19

**Authors:** Angeline Jeyakumar, Prasad Babar, Pramila Menon, Raji Nair, Suresh Jungari, Aishwarya Medhekar, Bhrunal Prakshale, Jasmine Shaikh, Merlin Chacko, Mohini Nikam, Purva More, Shakila Nayel, Similo Simelane, Sudeshna Awale

**Affiliations:** 1 School of Health Sciences, Savitribai Phule Pune University, Pune, Maharashtra India; 2 School of Tourism and Hospitality, University of Johannesburg, Johannesburg, South Africa; 3 Dr. D. Y. Patil Medical College Hospital and Research Centre, Pimpri Chinchwad, Maharashtra India; 4 UNICEF, Mumbai, India

**Keywords:** Complementary feeding, Urban slums, Critical age, Diet diversity, Minimum meal frequency, Minimum acceptable diet, Continued breastfeeding

## Abstract

**Background:**

Inequalities in child feeding practices are evident in urban slums in developing nations. Our study identified the determinants of complementary feeding (CF) practices in the informal settings of Pune, India, a district close to the business capital of India.

**Methods:**

Employing a cross-sectional study design, 1066 mother–children dyads were surveyed. Five indicators defined by the WHO were used to study complementary feeding practices. Determinants of complementary feeding practices were identified using multivariate analyses.

**Results:**

Timely initiation of CF was reported by 42%. Minimum acceptable diet (MAD), minimum meal frequency (MMF), and Diet Diversity Score > 4 were achieved by 14.9%, 76.5%, and 16.4%, respectively. Continued breastfeeding (CBF) at 2 years, and feeding processed foods were practiced by 94% and 50%, respectively. Among the maternal characteristics, a mother’s age > 30 years at pregnancy was less likely to achieve DD [AOR: 0.195 (CI 0.047–0.809)] and MAD [AOR: 0.231 (CI 0.056–0.960)]. Mothers with lower education were less likely to meet MMF [AOR: 0.302 (0.113–0.807)], MAD [AOR: 0.505 (CI 0.295–0.867)] and to introduce formula feeds (FF) [AOR: 0.417 (0.193- 0.899)]. Among obstetric characteristics, birth spacing < 33 months was less likely to achieve DD [AOR: 0.594 (CI 0.365–0.965)] and CBF [AOR: 0.562 (CI: 0.322–0.982)]. Receiving IYCF counseling only during postnatal care hindered the timely initiation of CF [AOR: 0.638 (0.415–0.981)]. Very Low Birth Weight increased the odds of achieving DD [AOR: 2.384 (1.007–5.644)] and MAD [AOR: 2.588(CI: 1.054–6.352)], while low birth weight increased the odds of children being introduced to processed foods [AOR: 1.370 (CI: 1.056–1.776)]. Concerning socio-economic status, being above the poverty line increased the odds of achieving MMF, [AOR: 1.851 (1.005–3.407)]. Other backward castes showed higher odds of achieving MAD [AOR: 2.191 (1.208–3.973)] and undisclosed caste in our study setting decreased the odds of FF [AOR: 0.339 (0.170–0.677)]. Bottle feeding interfered with MMF [AOR: 0.440 (0.317–0.611)] and CBF [AOR: 0.153 (0.105–0.224)].

**Conclusion:**

Investing in maternal education and IYCF counseling during both ANC and PNC to provide nutritious complementary foods alongside addressing poverty should be a national priority to prevent the double burden of undernutrition at an early age in informal settings.

## Background

Remarkable global gains in child survival are daunted by uneven progress particularly among the underprivileged in the developing world. Undernutrition remains a major determinant of child mortality across the globe. Worldwide, in 2019 an estimated 5.2 million children died before the age of five; of which 50% were due to undernutrition[1]. Global estimates suggest that over 50% of the global under-five population resides in Asia and Africa, and the worst impact of childhood undernutrition and its consequences are witnessed in these regions [2]. Among the 10 key ‘nutrition specific’ strategies to enable child growth and survival, infant and young child feeding (IYCF) practices have been promoted extensively [3]. It has been well established that optimal nutrition during the first two years in addition to lowering morbidity and mortality, reduces the risk of chronic disease and enables cognitive development [4, 5]. Despite global efforts to optimize IYCF, its progress has been relatively slow and suboptimal [1]. The introduction of complementary foods at 6 months of age, while continuing to breastfeed, is a global recommendation to enable age-appropriate growth and development. It is also one of the established interventions that can significantly reduce stunting during the first 2 years of life [6]. Evidence from developing countries suggests that poor complementary feeding practices could potentially risk a reduction in total energy and nutrient intake. Further, unhygienically prepared complementary food increases the risk of diarrheal infection and undernutrition among children 6–23 months of age [7, 8]. The diets of infants and children are often deficient in micronutrients such as iron, vitamin, zinc, and B vitamins in low-income countries. Thus, achieving adequacy in quantity and quality of complementary foods remains a challenge in the developing world.

In India, according to National Family Health Survey round 4 (NFHS-4) [9], less than 50% of children below two years of age received solid or semi-solid food and breastmilk, and less than 10% of children received an adequate diet. Consequently, India reports 50% child mortality, > 35% of stunting and underweight, and close to 30% wasting among children under five years [9], which calls for immediate attention. While a plethora of research on IYCF practices exists, community-based studies on complementary feeding practices employing WHO indicators, in vulnerable settings are limited. National surveys and existing regional studies with broad age categorization of children (0–5 years) identify the need to study children below two years, who are in the critical period of growth and development. Therefore, this work aimed to study complementary feeding practices and their determinants among children aged 6–24 months in Pune city.

## Methodology

### Study design and setting

A community-based cross-sectional survey was conducted between December 2018 and April 2019, in ten urban slums of Pune city, in Maharashtra, India. Pune is the second-largest city in the state after Mumbai [10]. The administrative division of Pune city comprises 15 wards. It is estimated that more than 40% of city dwellers live in the slums. These settings are characterized by poor living conditions, inadequate living space, poor access to safe drinking water and household and environmental sanitation and hygiene, and poor residential stability that reflects poor health indicators [11].

### Sample size and sampling technique

Multistage stratified systematic random sampling was used to recruit the participants. Out of the fifteen administrative wards of Pune Municipal Corporation (PMC), ten were selected using simple random sampling using an online random number generator. The population of each ward was further considered to select a population proportionate sample. Considering a prevalence of 48.8%, i.e., 0.48 of children age 6–8 months receiving complementary food (solid or semi-solid food and breastmilk) in urban Maharashtra, the sample size of the study was determined [9, 12]. Allowing 5% error and 95% confidence interval, 1.5 design effect, and 10% non-response, the sample size estimated was *n* = 633. For the major survey that included all IYCF indicators, to ensure representation of children aged 0–6 months for exclusive breastfeeding practices, the sample was doubled to *n* = 1443 after pre-test. From this work, a subsample of children aged 6–24 months (*n* = 1066) was considered. Children without congenital anomalies or chronic illness and their mothers who consented were considered for this study.

### Independent variables

The independent variables included maternal characteristics such as age at marriage and pregnancy, education, birth spacing, and type of delivery. Socio-demographic information such as family size, caste, and the color of ration cards as a proxy for economic status, and healthcare-related factors like IYCF counseling were also elicited. Child characteristics such as age, gender, term, and birth weight were recorded.

### Outcome variables

Complementary feeding (CF) indicators include its initiation in 6–8 months, Diet Diversity Score (DDS), minimum meal frequency (MMF), minimum acceptable diet (MAD), and optional indicators like Continued Breastfeeding at 2 years were dependent variables. Formula feeds and processed foods were additional variables in this study. A 24-h recall method was used to elicit information on dietary diversity and meal frequency from mothers and/or caregivers.

Dietary diversity score was assessed based on IYCF recommendation among seven food categories. The time of introduction of complementary foods and their adequacy were assessed as per the information provided by the mothers. Data were collected by researchers trained in public health nutrition.

### Statistical analysis

For data analysis, SPSS 20 was used. Descriptive statistics were computed for socio-demographic, maternal, and child variables. Multivariate analyses were performed to identify the determinants of complementary feeding practices. *P*-values < 0.05 were considered statistically significant.

### Ethical considerations

The study was approved by the institutional ethics committee of Savitribai Phule Pune University (SPPU/IEC/2019/06). Permission was taken from Pune and Pimpri Chinchwad Municipal Corporation. Procedures for the survey were designed to protect participants’ privacy by allowing for anonymous and voluntary participation. After explaining the objective, benefits, and risks of the study, written informed consent was signed by willing participants. The questionnaire was translated into the local language (Marathi).

## Results

A sample of 1066 mother–children dyads was included in this study after excluding the sample 0–6 months. Table 1 shows the frequency distribution of maternal and socio-demographic characteristics. Almost 10% of mothers were below 20 years of age and greater than 60% received secondary education. Family size > 4 was reported by 64.4%. Among the obstetric characteristics, parity up to two was reported by 82.5%. Normal delivery was reported by almost 68% and 64% were delivered in a public facility. Nearly 60% received advice for IYCF practices from healthcare workers. Of these, 40% received counseling during postnatal care.

**Table 1:** Distribution of socio-demographic and obstetric characteristics of respondents (*N* = 1066)

Maternal characteristics	*n*	%
*Maternal age (years)*		
< 20	102	9.6
21–25	536	50.3
26–30	347	32.6
> 30	81	7.6
*Maternal education*		
No formal education	47	4.4
Primary education	27	2.5
Secondary education	656	61.5
Higher secondary education	201	18.9
Graduation and above	135	12.7
*Religion*		
Hindu	718	67.4
Muslim	273	25.6
Others	75	7
*Caste*		
Open	289	27.1
Scheduled caste (SC)	306	28.7
Scheduled tribe (ST)	26	2.4
Other backward caste (OBC)	110	10.3
Not mentioned	335	31.4
*Maternal employment status*		
Not working	1010	94.7
Working	56	5.3
*Maternal employment during pregnancy*		
No	1024	96.1
Yes	42	3.9
*Number of family members*		
Up to 4	379	35.6
More than 4	687	64.4
*Number of children*		
Up to 2	879	82.5
More than 2	187	17.5
*Type of delivery*		
C-section	346	32.5
Normal	720	67.5
*Place of delivery*		
Public facility	680	63.8
Private facility	386	36.2
*Information received from health care providers*		
Yes	622	58.3
No	444	41.7
*Time of information*		
Antenatal care	80	7.5
Postnatal care	427	40.1
Both	115	10.8

Child characteristics identified mean age as 14.50 ± 4.86 months. Children born full term were 93%, while over 40% were either low or very low birth weight as per immunization schedule records. Both sexes were almost equally represented (Table 2).

**Table 2:** Distribution of child characteristics

Child characteristics	*n*	%
*Child’s age*		
Mean age (in months)	14.50 ± 4.86	
Median age (in months)	14	
6–8 months	140	13.1
9–24 months	926	86.9
*Gender*		
Male	523	49.1
Female	543	50.9
*Gestational age*		
Preterm (< 37 weeks)	75	7
Full-term (> 38 weeks)	991	93.0
*Birth weight*		
Very low birth weight (< 1500 gm)	34	3.2
Low birth weight (1500 gm–2500 gm)	418	39.2
Normal birth weight (< 2500 gm)	601	56.4
Not available	13	1.2

Table 3 shows the frequency distribution of the complementary feeding practices. A diet diversity score of 4 was not achieved by 84%. Nutrient quality of complementary feeds as assessed by MMF and MAD was reportedly achieved by 76.5 and 14.9%, respectively. Age-appropriate complementary feeding was initiated (AAICF) by less than one-half of the respondents, whereas breastfeeding was continued along with complementary foods by 94%. One-half of the participants reported having been given processed foods on the previous day of data collection.

**Table 3:** Distribution of prevalent complementary feeding practices

Complementary feeding practices	*n*	%
*Diet Diversity Score (DDS)*	2.61 ± 1.04	
< 4	891	83.6
6–8 months	133	95
9–11 months	184	89.3
12–24 months	574	79.7
> 4	175	16.4
6–8 months	7	5
9–11 months	22	10.7
12–24 months	146	20.3
*Minimum meal frequency (MMF)*	815	76.5
6–8 months	95	67.9
9–11 months	159	77.2
12–24 months	561	77.9
*Minimum acceptable diet (MAD)*	159	14.9
6–8 months	7	5
9–11 months	22	10.7
12–24 months	130	18.1
*Complementary feeding initiation age (months)*	6.52 ± 2.05	
6–8 months (timely)	450	42.2
< 6 months (early)	532	49.9
> 8 months (delayed)	59	5.5
Not yet initiated	25	2.3
*Formula feed was given the previous day*	89	8.3
6–8 months	24	17.1
9–11 months	24	11.7
12–24 months	41	5.7
*Processed food was given the previous day*	539	50.6
6–8 months	38	27.1
9–11 months	99	48.1
12–24 months	402	55.8
*Bottle feeding*	272	25.5
6–8 months	24	17.1
9–11 months	65	31.6
12–24 months	183	25.4
*Continued breastfeeding (CBF) at two years (20–24 months)*		
Yes	178	93.68
No	12	6.31

## Results of multivariate analysis

We examined the associations between complementary feeding practices and socio-demographic characteristics.

### Introduction of solid, semi-solid and soft foods (6–8 months) (ISSSF)

Factors associated with the introduction of solid, semi-solid and soft foods are presented in Table 4. Not receiving IYCF counselling showed less probability to impact *AAICF* according to both models [COR: 0.645 (CI 0.427–0.974); AOR 0.638 (CI 0.415–0.980)]. A similar inverse association was observed when counseling was received only during postnatal care (compared to during both antenatal and postnatal care) when adjusted with covariates [AOR: 0.638 (CI 0.415–0.981)]. VLBW was less likely to increase the odds of *AAICF* as per the crude model [COR: 0.440 (CI 0.198–0.980)] but was not significant in the adjusted model.

**Table 4:** Crude and adjusted odds ratio of socio-demographic, maternal, child characteristics with complementary feeding initiation in 6–8 months

Characteristics	Complementary feeding initiation in 6–8 months	
	Crude OR (95% CI)	Adjusted OR (95% CI)
*Mother's age at marriage*		
< 20^®^		
21–25	1.069 (0.780–1.463)	1.106 (0.789–1.550)
26–30	0.791 (0.364–1.718)	0.961 (0.415–2.226)
> 30	3.202 (0.259–39.60)	5.098 (0.392–66.34)
*Mother's age at last pregnancy*		
< 20^®^		
21–25	1.186 (0.848–1.657)	1.131 (0.780–1.639)
26–30	1.030 (0.686–1.547)	0.932 (0.567–1.531)
> 30	0.842 (0.411–1.729)	0.622 (0.265–1.459)
*Mother's education*		
No formal education	1.467 (0.725–2.969)	1.613 (0.761–3.422)
Primary education	0.747 (0.300–1.861)	0.910 (0.356–2.323)
Secondary education	1.456 (0.973–2.177)	1.492 (0.977–2.278)
Higher secondary education	1.345 (0.850–2.130)	1.357 (0.847–2.175)
Graduation and above^®^		
*Caste*		
Open^®^		
SC	1.165 (0.818–1.659)	1.167 (0.810–1.679)
ST	0.954 (0.414–2.197)	0.959 (0.410–2.240)
OBC	0.672 (0.422–1.068)	0.703 (0.436–1.134)
Not disclosed	0.787 (0.565–1.095)	0.771 (0.550–1.080)
*Religion*		
Hindu^®^		
Muslim	0.956 (0.700–1.304)	0.971 (0.703–1.342)
Others	1.133 (0.689–1.863)	1.122 (0.671–1.875)
*No of family members*		
< 4^®^		
> 4	0.864 (0.662–1.127)	0.875 (0.656–1.168)
*Color of ration card (proxy annual income in rupees)*		
Yellow (< 15,000)	1.400 (0.781–2.510)	1.299 (0.713–2.366)
Saffron (15,000–100,000)	1.713 (0.972–3.021)	1.644 (0.917–2.947)
White (> 100,000)^®^		
*Type of delivery*		
Normal^®^		
C-Section	0.979 (0.748–1.282)	1.041 (0.784–1.382)
*Place of delivery*		
Private facility^®^		
Public facility	1.138 (0.875–1.481)	1.155 (0.876–1.522)
*Birth spacing*		
Single child	0.826 (0.621–1.100)	0.754 (0.519–1.095)
< 33 months	0.954 (0.690–1.319)	0.890 (0.623–1.272)
> 33 months^®^		
*IYCF counselling received*		
Yes^®^		
No	0.645* (0.427–0.974)	0.638* (0.415–0.980)
*Time of IYCF counselling*		
Antenatal care	1.086 (0.614–1.923)	1.076 (0.592–1.954)
Postnatal care	0.676 (0.447–1.022)	0.638* (0.415–0.981)
Both^®^		
*Gender of child*		
Male^®^		
Female	0.890 (0.695–1.139)	0.894 (0.692–1.155)
*Gestational age*		
Preterm	1.384 (0.848–2.258)	1.456 (0.882–2.405)
Full term^®^		
*Birth weight of child*		
Very low (< 1500 gm)	0.440* (0.198–0.980)	0.477 (0.210–1.085)
Low (1500-2500gm)	0.937 (0.726–1.209)	0.947 (0.728–1.232)
Normal^®^ (> 2500 gm)		
*Birth order*		
< 2^®^		
> 2	0.849 (0.613–1.176)	0.800 (0.533–1.200)
*Bottle feeding*		
Yes	1.059 (0.801–1.400)	1.025 (0.762–1.379)
No^®^		

^®^ is the reference category, level of significance *p-value of < 0.05, **p-value of < 0.01, ***p-value of < 0.001

### Minimum diet diversity

Mother’s age at pregnancy > 30 years [AOR: 0.195 (CI 0.047–0.809)] was less likely to achieve DD in the adjusted model. Although family size > 4 [COR: 1.490 (CI 1.026–2.164), AOR: 1.440 (0.963–2.153)] increased the odds of achieving DD by 1.4 times, the difference was not significant after adjustment. One-third of the respondents (31%) did not disclose their caste. Caste had a significant impact on DD. Being OBC [COR: 2.060 (CI 1.178–3.605); AOR: 2.074 (CI 1.168–3.681)] increased the odds of achieving DD in both models by 2 times, compared to those in the socioeconomically better open category. Birth spacing less than 33 months [COR: 0.604 (0.385–0.949); AOR: 0.594 (CI 0.365–0.965)] was 40% less likely to achieve DD compared to spacing > 33 months in both models. In child characteristics, VLBW increased the odds of achieving diet diversity twice as compared to children with normal birth weight in the adjusted model [AOR: 2.384 (1.007–5.644)] (Table 5).

**Table 5:** Crude and adjusted odds ratio of socio-demographic, maternal, child characteristics with complementary feeding practices

Characteristics	Diet diversity		Minimum meal frequency		Minimum acceptable diet	
	Crude OR (95% CI)	Adjusted OR (95% CI)	Crude OR (95% CI)	Adjusted OR (95% CI)	Crude OR (95% CI)	Adjusted OR (95% CI)
*Mother's age at marriage*						
< 20^®^						
21–25	0.919 (0.604–1.397)	1.012 (0.644–1.592)	0.929 (0.641–1.346)	0.924 (0.618–1.382)	0.797 (0.513–1.241)	0.915 (0.568–1.475)
26–30	1.828 (0.746–4.483)	2.553 (0.940–6.932)	0.784 (0.330–1.860)	0.794 (0.305–2.064)	1.292 (0.495–3.376)	1.836 (0.632–5.336)
> 30	0–0	0–0	0–0	0–0	0–0	0–0
*Mothers age at last pregnancy*						
< 20^®^						
21–25	1.036 (0.655–1.639)	0.892 (0.536–1.482)	0.907 (0.613–1.341)	0.962 (0.621–1.490)	0.999 (0.624–1.601)	0.822 (0.486–1.390)
26–30	1.369 (0.802–2.338)	1.012 (0.522–1.964)	1.081 (0.670–1.745)	1.105 (0.613–1.990)	1.381 (0.798–2.390)	0.960 (0.486–1.896)
> 30	0.450 (0.141–1.441)	0.195* (0.047–0.809)	0.966 (0.427–2.184)	0.902 (0.344–2.361)	0.551 (0.173–1.755)	0.231* (0.056–0.960)
*Mother's education*						
No formal education	0.882 (0.356–2.187)	0.892 (0.346–2.296)	0.675 (0.292–1.562)	0.687 (0.275–1.717)	0.885 (0.355–2.205)	0.806 (0.310–2.092)
Primary education	0.650 (0.204–2.075)	0.542 (0.164–1.797)	0.305** (0.120–0.777)	0.302* (0.113–0.807)	0.648 (0.202–2.079)	0.498 (0.148–1.671)
Secondary education	0.662 (0.402–1.090)	0.593 (0.351–1.005)	0.603 (0.359–1.014)	0.584 (0.340–1.003)	0.604 (0.364–1.005)	0.505** (0.295–0.867)
Higher secondary education	1.005 (0.575–1.756)	0.991 (0.560–1.756)	0.628 (0.349–1.129)	0.636 (0.348–1.163)	0.769 (0.429–1.377)	0.725 (0.399–1.317)
Graduation and above^®^						
*Caste*						
Open^®^						
SC	1.185 (0.732–1.918)	1.156 (0.704–1.896)	1.477 (0.955–2.285)	1.436 (0.912–2.261)	1.231 (0.745–2.035)	1.183 (0.705–1.986)
ST	0.827 (0.231–2.962)	0.812 (0.223–2.960)	0.770 (0.310–1.912)	0.657 (0.260–1.663)	0.617 (0.137–2.782)	0.586 (0.128–2.690)
OBC	2.060** (1.178–3.605)	2.074** (1.168–3.681)	1.448 (0.822–2.549)	1.398 (0.782–2.499)	2.150** (1.206–3.833)	2.191** (1.208–3.973)
Not disclosed	1.046 (0.662–1.654)	0.984 (0.618–1.565)	0.843 (0.583–1.219)	0.786 (0.538–1.147)	1.124 (0.699–1.809)	1.054 (0.651–1.707)
*Religion*						
Hindu^®^						
Muslim	1.096 (0.724–1.659)	1.099 (0.716–1.687)	0.761 (0.539–1.074)	0.799 (0.556–1.146)	1.078 (0.701–1.657)	1.084 (0.695–1.691)
Others	1.019 (0.519–2.002)	1.097 (0.550–2.190)	0.947 (0.523–1.717)	1.207 (0.627–2.321)	0.997 (0.496–2.001)	1.080 (0.529–2.205)
*No of family members*						
< 4^®^						
> 4	1.490* (1.026–2.164)	1.440 (0.963–2.153)	1.237 (0.910–1.681)	1.225 (0.875–1.715)	1.479* (1.003–2.180)	1.395 (0.916–2.123)
*Color of ration card (proxy annual income in rupees)*						
Yellow (< 15,000)	0.748 (0.368–1.519)	0.705 (0.341–1.460)	1.767 (0.983–3.175)	1.524 (0.817–2.843)	0.892 (0.408–1.952)	0.852 (0.381–1.907)
Saffron (15,000–100,000)	0.744 (0.376–1.472)	0.692 (0.343–1.395)	2.203** (1.244–3.900)	1.851* (1.005–3.407)	0.987 (0.464–2.099)	0.935 (0.430–2.031)
White (> 100,000)^®^						
*Type of delivery*						
Normal^®^						
C-Section	1.130 (0.793–1.610)	1.139 (0.783–1.655)	1.073 (0.783–1.469)	1.116 (0.797–1.562)	1.112 (0.769–1.610)	1.127 (0.762–1.666)
*Place of delivery*						
Private facility^®^						
Public facility	1.013 (0.715–1.435)	1.076 (0.744–1.557)	1.083 (0.799–1.469)	1.173 (0.848–1.623)	1.099 (0.762–1.585)	1.166 (0.792–1.717)
*Birth spacing*						
Single child	0.822 (0.569–1.187)	0.708 (0.434–1.157)	0.968 (0.691–1.355)	0.992 (0.634–1.553)	0.743 (0.506–1.090)	0.676 (0.405–1.129)
< 33 months	0.604* (0.385–0.949)	0.594* (0.365–0.965)	0.843 (0.579–1.227)	0.864 (0.566–1.319)	0.626* (0.395–0.991)	0.630 (0.384–1.035)
> 33 months^®^						
*IYCF counselling received*						
Yes^®^						
No	1.198 (0.667–2.150)	1.276 (0.691–2.354)	0.782 (0.475–1.287)	0.826 (0.485–1.405)	1.227 (0.673–2.236)	1.268 (0.677–2.375)
*Time of IYCF counselling*						
Antenatal care	1.796 (0.853–3.782)	1.902 (0.869–4.164)	1.359 (0.645–2.863)	1.463 (0.656–3.263)	1.935 (0.910–4.118)	2.068 (0.935–4.572)
Postnatal care	1.193 (0.663–2.146)	1.243 (0.675–2.289)	0.840 (0.509–1.388)	0.796 (0.467–1.357)	1.027 (0.558–1.890)	1.035 (0.550–1.950)
Both^®^						
*Gender of child*						
Male^®^						
Female	0.934 (0.673–1.298)	0.953 (0.677–1.340)	1.049 (0.786–1.399)	1.076 (0.794–1.458)	0.833 (0.591–1.173)	0.831 (0.582–1.185)
*Gestational age*						
Preterm	0.962 (0.506–1.830)	0.965 (0.497–1.875)	1.065 (0.599–1.892)	1.074 (0.587–1.967)	0.883 (0.445–1.755)	0.898 (0.441–1.827)
Full term^®^						
Birth weight of child						
Very low (< 1500 gm)	2.081 (0.915–4.731)	2.384* (1.007–5.644)	0.878 (0.380–2.025)	1.283 (0.529–3.114)	2.085 (0.886–4.904)	2.588*(1.054–6.352)
Low (1500-2500gm)	1.183 (0.843–1.660)	1.268 (0.892–1.804)	0.815 (0.607–1.096)	0.917 (0.673–1.250)	1.145 (0.804–1.630)	1.254 (0.868–1.811)
Normal^®^ (> 2500 gm)						
*Birth order*						
< 2^®^						
> 2	1.190 (0.786–1.802)	1.142 (0.673–1.938)	0.995 (0.683–1.450)	0.909 (0.564–1.467)	1.306 (0.856–1.993)	1.133 (0.659–1.946)
*Bottle feeding*						
Yes	1.024 (0.705–1.487)	0.922 (0.621–1.369)	2.195*** (1.619–2.977)	0.440*** (0.317–0.611)	1.202 (0.806–1.793)	0.802 (0.526–1.224)
No^®^						

^®^ is the reference category, level of significance *p-value of < 0.05, **p-value of < 0.01, ***p-value of < 0.001

### Minimum meal frequency (MMF)

Mother’s education up to primary level [COR: 0.305 (CI 0.120–0.777); AOR: 0.302 (CI 0.113–0.807)] was less likely to achieve MMF as per the crude and adjusted model. Economic status above the poverty line increased the odds of achieving MMF, [COR: 2.203 (CI 1.244–3.900), AOR: 1.851 (1.005–3.407)]. Bottle feeding [COR: 2.195 (CI 1.619–2.977); AOR: 0.440 (CI 0.317–0.611)] increased the odds of meeting MMF by two times compared to not bottle feeding in the crude model. However, the relationship was inverse and significant in the adjusted model (Table 5).

### Minimum acceptable diet (MAD)

As per the adjusted model, the mother’s age at pregnancy above 30 years was less likely to meet the requirements of MAD [AOR: 0.231 (CI 0.056–0.960)]. The odds of providing MAD were 50% lesser among mothers who received education up to secondary level than up to graduation and above [AOR: 0.505 (CI 0.295–0.867)]. Belonging to the backward caste (OBC) increased the odds of achieving MAD two times in both models [COR: 2.150 (CI: 1.206–3.833), AOR: 2.191 (1.208–3.973)] as compared to those who belonged to the socioeconomically better open category as per the caste categorization. Family size > 4 increased the odds of receiving MAD as per the crude model by 1.4 times [COR: 1.479 (CI: 1.003–2.180)], which was not significant after adjustment, whereas VLBW increased the odds of receiving MAD by 2.5 times in the adjusted model [AOR: 2.588(CI: 1.054–6.352)] (Table 5).

### Formula feeding (FF)

Mothers with education up to higher-secondary level were 59% less likely to practice FF [COR: 0.387 (CI: 0.183–0.821); AOR: 0.417 (0.193–0.899)], compared to education up to graduation or higher. Undisclosed caste decreased the odds of FF in both models [COR: 0.374 (CI 0.191–0.731); AOR: 0.339 (0.170–0.677)]. Birth order > 2 [COR: 0.441 (0.209–0.931)] and bottle feeding [COR: 0.580 (CI 0.367- 0.916)] was less likely to impact introducing formula feeds as per the crude model, however, the association was not significant in the adjusted model (Table 6).

**Table 6:** Crude and adjusted odds ratio of socio-demographic, maternal, child characteristics with formula feeding

Characteristics	Formula feed	
	Crude OR (95% CI)	Adjusted OR (95% CI)
*Mother's age at marriage*		
< 20^®^		
21–25	1.262 (0.729–2.187)	1.070 (0.589–1.943)
26–30	1.448 (0.421–4.978)	0.936 (0.247- 3.541)
> 30	0–0	0–0
*Mother's age at last pregnancy*		
< 20^®^		
21–25	0.815 (0.444–1.496)	0.947 (0.488–1.837)
26–30	0.861 (0.418–1.772)	1.307 (0.539–3.172)
> 30	0.596 (0.149–2.376)	0.898 (0.187–4.323)
*Mother's education*		
No formal education	0.157 (0.020–1.240)	0.247 (0.031–2.002)
Primary education	0.226 (0.029–1.790)	0.247 (0.030–2.042)
Secondary education	0.578 (0.324–1.031)	0.663 (0.359–1.225)
Higher secondary education	0.387** (0.183–0.821)	0.417* (0.193- 0.899)
Graduation and above^®^		
*Caste*		
Open^®^		
SC	0.985 (0.550–1.762)	1.037 (0.566–1.900)
ST	0.636 (0.138–2.924)	0.679 (0.146–3.162)
OBC	0.593 (0.259–1.357)	0.544 (0.234–1.268)
Not disclosed	0.374** (0.191–0.731)	0.339** (0.170–0.677)
*Religion*		
Hindu^®^		
Muslim	0.847 (0.464–1.548)	0.918 (0.492–1.713)
Others	1.170 (0.493–2.777)	0.936 (0.366–2.391)
No of family members		
< 4^®^		
> 4	1.053 (0.648–1.708)	1.131 (0.676–1.891)
*Color of ration card (proxy annual income in rupees)*		
Yellow (< 15,000)	0.920 (0.331–2.556)	0.902 (0.318–2.563)
Saffron (15,000–100,000)	0.919 (0.344–2.459)	0.902 (0.330–2.469)
White (> 100,000)^®^		
*Type of delivery*		
Normal^®^		
C-Section	0.978 (0.609–1.572)	0.935 (0.567–1.542)
*Place of delivery*		
Private facility^®^		
Public facility	0.754 (0.478–1.189)	0.773 (0.476–1.256)
*Birth spacing*		
Single child	1.573 (0.944–2.622)	1.317 (0.676–2.567)
< 33 months	0.791 (0.405–1.542)	0.815 (0.391–1.701)
> 33 months^®^		
*IYCF counselling received*		
Yes^®^		
No	1.279 (0.552–2.966)	1.867 (0.735- 4.746)
*Time of IYCF counselling*		
Antenatal care	1.956 (0.697–5.490)	2.581 (0.833–7.998)
Postnatal care	1.551 (0.675–3.565)	1.941 (0.773–4.874)
Both^®^		
*Gender of child*		
Male^®^		
Female	1.036 (0.666–1.611)	1.065 (0.672–1.690)
*Gestational age*		
Preterm	0.736 (0.282–1.919)	0.657 (0.244–1.770)
Full term^®^		
Birth weight of child		
Very low (< 1500 gm)	1.412 (0.463–4.302)	1.464 (0.452–4.740)
Low (1500-2500gm)	0.825 (0.517–1.314)	0.857 (0.528–1.390)
Normal^®^ (> 2500 gm)		
*Birth order*		
< 2^®^		
> 2	0.441* (0.209–0.931)	0.559 (0.232–1.349)
*Bottle feeding*		
Yes	0.580* (0.367- 0.916)	1.574 (0.970–2.554)
No^®^		

^®^ is the reference category, level of significance *p-value of < 0.05, **p-value of < 0.01, ***p-value of < 0.001

### Processed foods

Children in Muslim families were more likely to receive processed foods; however, this association was not significant after adjustment. [COR: 1.394 (CI 1.027–1.891), AOR: 1.329 (0.969–1.822)]. According to both models, low birth weight increased the odds of children being introduced to processed foods by 1.3 times [COR: 1.351 (CI: 1.049–1.741) AOR: 1.370 (CI: 1.056–1.776)] (Table 7).

**Table 7:** Crude and adjusted odds ratio of socio-demographic, maternal, child characteristics with processed food feeding

Characteristics	Processed food feeding	
	Crude OR (95% CI)	Adjusted OR (95% CI)
*Mother's age at marriage*		
< 20^®^		
21–25	1.091 (0.799–1.489)	1.085 (0.778–1.515)
26–30	0.924 (0.441–1.935)	0.885 (0.395–1.983)
> 30	1.893 (0.157–22.86)	2.350 (0.184–30.00)
*Mother's age at last pregnancy*		
< 20^®^		
21–25	0.995 (0.716–1.384)	0.943 (0.655–1.357)
26–30	1.088 (0.729–1.622)	1.028 (0.632–1.673)
> 30	1.090 (0.544–2.185)	1.056 (0.466–2.397)
*Mother's education*		
No formal education	1.056 (0.527–2.114)	0.850 (0.406–1.778)
Primary education	1.111 (0.479–2.574)	1.070 (0.450–2.545)
Secondary education	1.326 (0.897–1.960)	1.214 (0.807–1.827)
Higher secondary education	1.177 (0.752–1.843)	1.106 (0.699–1.748)
Graduation and above^®^		
*Caste*		
Open^®^		
SC	1.062 (0.748–1.508)	1.075 (0.750–1.542)
ST	2.183 (0.904–5.274)	2.280 (0.937–5.550)
OBC	0.641 (0.407–1.008)	0.642 (0.402–1.023)
Not disclosed	0.862 (0.623–1.194)	0.850 (0.611–1.183)
*Religion*		
Hindu^®^		
Muslim	1.394* (1.027–1.891)	1.329 (0.969–1.822)
Others	0.846 (0.516–1.385)	0.850 (0.511–1.415)
*No of family members*		
< 4^®^		
> 4	1.200 (0.923–1.561)	1.163 (0.876–1.545)
*Color of ration card (proxy annual income in rupees)*		
Yellow (< 15,000)	0.995 (0.571–1.733)	1.026 (0.579–1.818)
Saffron (15,000–100,000)	0.990 (0.577–1.699)	1.044 (0.598–1.823)
White (> 100,000)^®^		
*Type of delivery*		
Normal^®^		
C-Section	0.903 (0.692–1.177)	0.924 (0.699–1.220)
*Place of delivery*		
Private facility^®^		
Public facility	0.961 (0.742–1.246)	0.919 (0.701–1.206)
*Birth spacing*		
Single child	0.903 (0.681–1.197)	0.971 (0.671–1.405)
< 33 months	1.229 (0.890–1.697)	1.217 (0.854–1.734)
> 33 months^®^		
*IYCF counselling received*		
Yes^®^		
No	1.028 (0.682–1.549)	1.002 (0.652–1.538)
*Time of IYCF counselling*		
Antenatal care	1.201 (0.678–2.129)	1.146 (0.630–2.084)
Postnatal care	0.951 (0.630–1.436)	0.928 (0.605–1.425)
Both^®^		
*Gender of child*		
Male^®^		
Female	1.160 (0.908–1.481)	1.176 (0.914–1.513)
*Gestational age*		
Preterm	1.404 (0.858–2.298)	1.434 (0.866–2.375)
Full term^®^		
*Birth weight of child*		
Very low (< 1500 gm)	0.773 (0.377–1.585)	0.785 (0.374–1.649)
Low (1500-2500gm)	1.351* (1.049–1.741)	1.370* (1.056–1.776)
Normal^®^ (> 2500 gm)		
*Birth order*		
< 2^®^		
> 2	1.377 (0.998–1.900)	1.147 (0.769–1.711)
*Bottle feeding*		
Yes	1.072 (0.814–1.412)	0.943 (0.705–1.261)
No^®^		

^®^ is the reference category, level of significance *p-value of < 0.05, **p-value of < 0.01, ***p-value of < 0.001

### Continued breastfeeding (CBF) at 2 years

Mothers who were more than 30 years old at marriage were less likely to continue breastfeeding their children at 2 years [COR: 0.066 (CI: 0.005–0.883), AOR: 0.129 (0.007–2.241)], in the crude but not in the adjusted model. Similarly, mothers' age at pregnancy between 26 and30 years and belonging to the scheduled caste increased the likelihood of CBF by 1.9 and 2.4 times, respectively, in the crude but not in the adjusted model. Muslims were less likely to CBF, according to the adjusted model [AOR: 0.790 (CI: 0.509–1.228)]. Single children were less likely to be given CBF as per COR, which was not significant in the adjusted model. Birth spacing of < 33 months was less likely to be given CBF as per AOR [AOR: 0.562 (CI: 0.322–0.982)]. Bottle-fed children were less likely to be continued BF as per both models [COR: 0.154 (CI: 0.109–0.219)] (Table 8).

**Table 8:** Crude and adjusted odds ratio of socio-demographic, maternal, and child characteristics with continued breastfeeding

Characteristics	Crude OR (95% CI)	Adjusted OR (95% CI)
*Mother's age at marriage*		
< 20^®^		
21–25	0.863 (0.558–1.335)	0.970 (0.592–1.591)
26–30	0.506 (0.201–1.277)	0.793 (0.267–2.356)
> 30	0.066* (0.005–0.883)	0.129 (0.007–2.241)
*Mother's age at last pregnancy*		
< 20^®^		
21–25	1.412 (0.913–2.184)	1.325 (0.787–2.231)
26–30	1.917* (1.105–3.326)	1.521 (0.740–3.128)
> 30	1.483 (0.591–3.724)	1.063 (0.332–3.406)
*Mother's education*		
No formal education	0.876 (0.354–2.168)	0.706 (0.248–2.013)
Primary education	0.403 (0.151–1.073)	0.383 (0.123–1.195)
Secondary education	1.068 (0.627–1.820)	0.914 (0.509–1.642)
Higher secondary education	1.089 (0.587–2.021)	1.045 (0.534–2.046)
Graduation and above^®^		
*Caste*		
Open^®^		
SC	2.439** (1.468–4.054)	2.362 (1.352–4.126)
ST	3.271 (0.734–14.58)	2.334 (0.492–11.07)
OBC	1.368 (0.749–2.498)	1.353 (0.697–2.626)
Not disclosed	1.359 (0.897–2.060)	1.342 (0.850–2.120)
*Religion*		
Hindu^®^		
Muslim	0.761 (0.515–1.125)	0.790** (0.509–1.228)
Others	0.643 (0.335–1.234)	0.618 (0.300–1.274)
*No of family members*		
< 4^®^		
> 4	0.944 (0.660–1.352)	1.133 (0.749–1.714)
*Color of ration card (proxy annual income in rupees)*		
Yellow (< 15,000)	1.564 (0.768–3.185)	1.216 (0.542–2.732)
Saffron (15,000–100,000)	1.559 (0.781–3.112)	1.109 (0.503–2.442)
White (> 100,000)^®^		
*Type of delivery*		
Normal^®^		
C-Section	1.118 (0.778–1.608)	1.217 (0.808–1.832)
*Place of delivery*		
Private facility^®^		
Public facility	1.383 (0.978–1.956)	1.377 (0.931–2.037)
*Birth spacing*		
Single child	0.556** (0.371–0.833)	0.593 (0.335–1.050)
< 33 months	0.728 (0.455–1.165)	0.562* (0.322–0.982)
> 33 months^®^		
*IYCF counselling received*		
Yes^®^		
No	0.650 (0.353–1.197)	1.433 (0.726–2.829)
*Time of IYCF counselling*		
Antenatal care	0.785 (0.342–1.802)	0.889 (0.350–2.262)
Postnatal care	0.745 (0.402–1.380)	0.859 (0.433–1.705)
Both^®^		
*Gender of child*		
Male^®^		
Female	0.935 (0.670–1.303)	0.963 (0.664–1.396)
*Gestational age*		
Preterm	1.368 (0.676–2.767)	1.379 (0.630–3.020)
Full term^®^		
*Birth weight of child*		
Very low (< 1500 gm)	0.636 (0.262–1.544)	1.162 (0.411–3.289)
Low (1500-2500gm)	0.816 (0.580–1.147)	0.840 (0.574–1.229)
Normal^®^ (> 2500 gm)		
*Birth order*		
< 2^®^		
> 2	1.198 (0.762–1.884)	0.753 (0.409–1.387)
*Bottle feeding*		
Yes	0.154*** (0.109–0.219)	0.153*** (0.105–0.224)
No^®^		
*Formula feed*		
Yes	0.880 (0.492–1.572)	0.809 (0.437–1.499)
No^®^		
*Processed food*		
Yes	1.339 (0.941–1.905)	1.449 (0.999–2.103)
No^®^		

^®^ is the reference category, level of significance *p-value of < 0.05, **p-value of < 0.01, ***p-value of < 0.001

## Discussion

Improving complementary feeding practices provides remarkable improvements in the nutritional status of children. Despite its established benefits, developing nations, especially populations living in informal settings rarely meet the requirements. The disparities evident in the national and regional statistics in the CF practices resulted in the search for setting specific determinants in the urban slums of Maharashtra. Urban slums constitute 10% of the population in Maharashtra that accounts for 1.18 crores and significantly add to the health statistics of the state [13]. Our work contributes to the scarce evidence that could provide valuable lessons for similar settings. We covered a representative sample from different slums with comprehensive detailing of CF practices employing the seven WHO indicators. Furthermore, we studied the introduction of infant formulas and processed foods, relevant for the ensuing double burden of malnutrition in underprivileged settings.

Age-appropriate initiation of CF has long-term health benefits throughout the life cycle [14]. Growth faltering that marks the onset of undernutrition, is a result of non-initiation of timely CF. Our study identified the low prevalence of timely initiation (< 50%) which is consistent with the national and Maharashtra state statistics [9, 15]. North-East region in India as an exception showed high prevalence. Poor knowledge among mothers is a proven determinant for poor IYCF practices. Response to interventions might vary between settings substantially. Our study identified that receiving counseling better impacted CF practices. Likewise, antenatal and immediate postnatal counseling showed a significant positive association in previous research [16, 17].

A minimum acceptable diet (MAD) is a direct indicator that correlates with nutritional status. In our study, 85% did not meet MAD requirements. Age of the children is known to impact MAD in other developing countries, where older children achieved MAD, compared to younger children [18, 19]. Similar were the observations in NFHS 3 and 4 [9, 20, 21]. Likewise the younger age group of our sample showed a high prevalence of non-adherence to MAD requirements. Among the maternal characteristics, mother’s age < 30 years, education [COR: 0.066 (CI: 0.005–0.883), AOR: 0.129 (0.007–2.241)] up to graduation or above, and higher societal position reflected by caste, were factors that enabled to meet MAD requirements. While the association of wealth status of mothers with MAD aligned with few studies, the higher age of mothers favored MAD in others [8, 22, 23]. In our study family size > 4 favored meeting MAD. Similar findings are observed in studies where a joint family system and higher birth order enabled MAD achievement [21, 24].

Minimum meal frequency (MMF) has been emphasized to provide small frequent meals to meet the nutritional requirements of children. Three-fourth of our participants could meet the MMF requirements which compares much higher than the Global Nutrition Report (41.9%) [25]. Among the South East Asian countries MMF was high in Maldives and Nepal comparable to our results [26, 27]. These observations reflect variations in socio-cultural practices and the economic status of the participants. Although in this study, the number of meals was recorded, the constituents of each meal were not elicited to derive the quality of the meal. Preparing and feeding frequent meals in resource-poor settings are determined by multiple factors. Our work identified low maternal education and economic status below the poverty line impede attaining MMF standards similar to other studies [28, 29]. Existing literature suggests that factors such as younger age [23]  bottle feeding diarrhea and respiratory tract infections prevented MMF [8]. In our work too children who were bottle-fed were less likely to meet MMF. In contrast to this, a study in Ethiopia reported high  MMF, despite extensive bottle feeding [30]. This could be explained by the variation in the intensity of bottle feeding and the encouragement to empty bottles practiced by the mothers in different settings [31].

Diet diversity (DD) greater than four was not achieved by mothers above 30 years of age in our study. Similar results were observed in Pakistan [32]. Heterogenous associations emerge between maternal age and child care in different settings. Khan’s [21] and Dhami’s [8] work reported that mothers' younger in age did not meet DD requirements. These were analyses of national surveys, compared to our work in a specific informal setting. Teenage mothers and mothers above 30 years showed evidence of poor complementary feeding practices. While young age is associated with poor knowledge and awareness, older age could probably reflect rigid dietary practices [32, 33]. Our study also showed VLBW infants had significantly higher diet diversity. In the absence of guidelines for complementary feeding practices for preterm/VLBW children, [34, 35] achieving diet diversity could be a challenge for mothers in informal settings. In our population, initial facility-based care owing to the VLBW, maternal awareness, and early weaning could perhaps have contributed to better diet diversity.

Formula feeds were less likely to be introduced by mothers who were less educated in our study sample. Evidence from 18 Low and Middle-Income Countries (LMIC) using 319 nationally representative surveys showed improved IYCF practices among women with higher education. However, the use of formula feeds was also higher among women with higher education in all regions [36]. The IYCF 2008 guidelines [37] did not include milk-based formulas. However, according to the 2021 guidelines [38] a criterion of ‘mixed milk feeding’ is added, which includes infant formulas or animal milk. But these are specific for children under 6 months. Although India adopted the Infant Milk Substitute Act in 1992, the market for infant formulas is predicted to increase in India, as observed in other countries indicating increased use of formulas [39].

Processed foods intake among children under two years is relevant for the rising double burden in India. In our work, LBW infants showed higher odds of taking processed foods. LBW is typically associated with poor maternal nutrition, in lower wealth quartiles. In informal settings poor access to healthy foods and increased access to unhealthy processed foods have been well documented [40, 41].

*Continued breastfeeding (CBF)* Traditional families reportedly continued breastfeeding in national surveys, which agrees with our findings where the Hindu religion observed CBF [42]. Parity influenced CBF in our study, where single children were continually breastfed, similar to findings from Sharma’s work [43]. Bottle feeding interfered with CBF in our study. Similar associations were observed with formula feeding and duration of BF in hospital-based studies [44, 45]. CBF widely practiced in India saw a decline from the 1990s to early 2000 [42]. These observations point to the child care transition alongside changing lifestyles in a developing nation.

Our study had certain important limitations. The cross-sectional study design limits deriving causality. The complementary feeding practices were reported by the mothers and not measured. Diet diversity did not derive information about the quality of the diet that limits interpretations. The urban slums in this region are likely to differ from other settings that limit generalizability. Despite these limitations, the seven indicators of complementary feeding identified clear patterns of association between specific maternal, economic, and cultural factors. Maternal age, education, and awareness, economic status, tradition, and religion determined CF. Children in these settings would perhaps begin their early years with nutritional deficits adding to the national burden and contributing to the DALY. Identifying setting specific determinants reveal opportunities to improve CF in urban slums.

## Conclusion

Our study highlights the need for interventions to address the prevalent poor complementary feeding practices. Investing in strategies to improve maternal awareness, on the importance of hygiene maintenance, harmful effect of processed food, and bottle feeding, through ante and postnatal services could positively impact the dietary intake of the family that would benefit young children. Immediate action involving multiple stakeholders to prevent access to processed foods would particularly ensure healthier complementary feeding in informal settings.

## Data Availability

Data will be available on request to the corresponding author.

## References

[1] Malnutrition in Children—UNICEF DATA [Internet]. [cited 2021 Nov 18]. Available from: https://data.unicef.org/topic/nutrition/malnutrition/

[2] Children: improving survival and well-being [Internet]. [cited 2021 Nov 18]. Available from: https://www.who.int/news-room/fact-sheets/detail/children-reducing-mortality

[3] Bhutta ZA, Das JK, Rizvi A, Gaffey MF, Walker N, Horton S (2013). Evidence-based interventions for improvement of maternal and child nutrition: what can be done and at what cost?. Lancet.

[4] Bhutta ZA, Berkley JA, Bandsma RHJ, Kerac M, Trehan I, Briend A (2017). Severe childhood malnutrition. Nat Rev Dis Prim.

[5] Jolly R (2007). Early childhood development: the global challenge. Lancet.

[6] Dewey KG, Adu-Afarwuah S (2008). Systematic review of the efficacy and effectiveness of complementary feeding interventions in developing countries. Maternal Child Nutr.

[7] Khanal V, Sauer K, Zhao Y (2013). Determinants of complementary feeding practices among Nepalese children aged 6–23 months: findings from demographic and health survey 2011. BMC Pediatr.

[8] Dhami MV, Ogbo FA, Osuagwu UL, Agho KE (2019). Prevalence and factors associated with complementary feeding practices among children aged 6–23 months in India: a regional analysis. BMC Public Health.

[9] International Institute for Population Sciences (IIPS) and ICF. National family health survey (NFHS-4) 2015–16 India [Internet]. Mumbai; 2017. Available from: http://www.rchiips.org/nfhs

[10] Registrar General and Census Commissioner I. Ranking of Districts by Population Density, 2001 and 2011 Report : [Internet]. [cited 2021 Nov 18]. Available from: https://data.maharashtra.gov.in/SDB_Reports/Population_Census/PDF/Ranking%20of%20Districts%20by%20Population%20Density,%202001%20and%202011.pdf

[11] Sanitation [Internet]. World Health Organization. 2021 [cited 2021 Nov 18]. Available from: https://www.who.int/news-room/fact-sheets/detail/sanitation

[12] Irving D, Daniel WW (1975). Biostatistics: a foundation for analysis in the health sciences. J R Stat Soc Ser A (Gen).

[13] Report of the committee on slum statistics/Census [Internet]. New Delhi; 2010 [cited 2021 Nov 18]. Available from: https://mohua.gov.in/upload/uploadfiles/files/9Slum_Report_NBO(2).pdf

[14] Przyrembel H (2012). Timing of the introduction of complementary food: short- and long-term health consequences. Annals Nutr Metabol.

[15] Menon P, Bamezai A, Subandoro A, Ayoya MA, Aguayo V (2015). Age-appropriate infant, and young child feeding practices are associated with child nutrition in India: insights from nationally representative data. Maternal Child Nutr.

[16] Namasivayam V, Dehury B, Prakash R, Becker M, Avery L, Sankaran D, et al. Association of prenatal counseling and immediate postnatal support with early initiation of breastfeeding in Uttar Pradesh, India. Int Breastfeed J [Internet]. 2021 Dec 1 [cited 2021 Nov 18];16(1). Available from: https://pubmed.ncbi.nlm.nih.gov/33726797/10.1186/s13006-021-00372-6 PMC7968284 33726797

[17] Rao S, Swathi PM, Unnikrishnan B, Hegde A (2011). Study of complementary feeding practices among mothers of children aged six months to two years—a study from coastal south India. Australas Med J.

[18] Tegegne M, Sileshi S, Benti T, Teshome M, Woldie H. Factors associated with minimal meal frequency and dietary diversity practices among infants and young children in the predominantly agrarian society of Bale zone, Southeast Ethiopia: a community based cross-sectional study. Arch Public Health [Internet]. 2017 Nov 13 [cited 2021 Nov 18];75(1). Available from: https://pubmed.ncbi.nlm.nih.gov/29158896/10.1186/s13690-017-0216-6 PMC5682638 29158896

[19] Kambale RM, Ngaboyeka GA, Kasengi JB, Niyitegeka S, Cinkenye BR, Baruti A (2021). Minimum acceptable diet among children aged 6–23 months in South Kivu, Democratic Republic of Congo: a community-based cross-sectional study. BMC Pediatr.

[20] National Family Health Survey (NFHS-3), 2005–06: India: Volume I. [Internet]. Mumbai; 2007 [cited 2021 Nov 18]. Available from: https://dhsprogram.com/pubs/pdf/frind3/frind3-vol1andvol2.pdf

[21] Khan N, Mozumdar A, Kaur S (2019). Dietary adequacy among young children in India: improvement or stagnation? An investigation from the national family health survey. Food Nutr Bull.

[22] Acharya A, Pradhan MR, Das AK (2021). Determinants of minimum acceptable diet feeding among children aged 6–23 months in Odisha, India. Public Health Nutr.

[23] Patel A, Pusdekar Y, Badhoniya N, Borkar J, Agho KE, Dibley MJ (2012). Determinants of inappropriate complementary feeding practices in young children in India: secondary analysis of National Family Health Survey 2005–2006. Maternal Child Nutr.

[24] Jain S, Bhan B, Bhatt G (2020). Complementary feeding practices and their determinants among children 6–23 months of age in an outpatient hospital setting in Central India: a cross-sectional study. J Fam Med Prim Care.

[25] Global Nutrition Report|Country Nutrition Profiles, India—Global Nutrition Report [Internet]. 2020 [cited 2021 Nov 18]. Available from: https://globalnutritionreport.org/resources/nutrition-profiles/asia/southern-asia/india/

[26] Aguayo VM. Complementary feeding practices for infants and young children in South Asia. A review of the evidence for action post-2015. Maternal Child Nutr [Internet]. 2017 Oct 1 [cited 2021 Nov 18]; 13(Suppl 2). Available from: https://pubmed.ncbi.nlm.nih.gov/29032627/10.1111/mcn.12439 PMC6865921 29032627

[27] Kabir I, Khanam M, Agho KE, Mihrshahi S, Dibley MJ, Roy SK (2012). Determinants of inappropriate complementary feeding practices in infant and young children in Bangladesh: secondary data analysis of Demographic Health Survey 2007. Maternal Child Nutr.

[28] Manikam L, Prasad A, Dharmaratnam A, Moen C, Robinson A, Light A (2018). Systematic review of infant and young child complementary feeding practices in South Asian families: the India perspective. Public Health Nutri.

[29] Houghton LA, McIntosh DR, Trilok-Kumar G, Haszard JJ, Gibson RS. Suboptimal feeding and caring practices among young Indian children ages 12 to 24 months living in the slums of New Delhi. Nutrition (Burbank, Los Angeles County, Calif) [Internet]. 2020 Jan 1 [cited 2021 Nov 18];69. Available from: https://pubmed.ncbi.nlm.nih.gov/31539814/10.1016/j.nut.2019.110553 31539814

[30] Mekonnen M, Kinati T, Bekele K, Tesfa B, Hailu D, Jemal K (2021). Infant, and young child feeding practice among mothers of children age 6 to 23 months in Debrelibanos district, North Showa Zone, Oromia region, Ethiopia. PLoS ONE.

[31] Ventura AK, Garcia P, Schaffner AA (2017). Associations between bottle-feeding intensity and maternal encouragement of bottle-emptying. Public Health Nutr.

[32] Ali M, Arif M, Shah AA (2021). Complementary feeding practices and associated factors among children aged 6–23 months in Pakistan. PLoS ONE.

[33] Lodha BV (2021). Assessment of complementary feeding practices and misconceptions regarding foods in young mothers. Int J Food Nutr Sci.

[34] Liotto N, Cresi F, Beghetti I, Roggero P, Menis C, Corvaglia L (2020). Complementary feeding in preterm infants: a systematic review. Nutrients.

[35] Baldassarre ME, Giannì ML, di Mauro A, Mosca F, Laforgia N (2020). Complementary feeding in preterm infants: where do we stand?. Nutrients.

[36] Neves PAR, Barros AJD, Gatica-Domínguez G, Vaz JS, Baker P, Lutter CK (2021). Maternal education and equity in breastfeeding: trends and patterns in 81 low- and middle-income countries between 2000 and 2019. Int J Equity Health.

[37] Indicators for assessing infant and young child feeding practices: definitions and measurement methods [Internet]. [cited 2021 Nov 18]. Available from: https://www.who.int/publications/i/item/9789240018389

[38] WHO. World Health Organization and the United Nations Children’s Fund (UNICEF). Indicators for assessing infant and young child feeding practices: definitions and measurement methods. Geneva: 2021

[39] Ministry of Law and Justice G of I. The Infant Milk Substitutes, Feeding Bottles, and Infant Foods (Regulation of Production, Supply, and Distribution) Act, 1992 [Internet]. 1992 [cited 2021 Nov 20]. Available from: https://legislative.gov.in/sites/default/files/A1992-41.pdf.

[40] Vilar-Compte M, Burrola-Méndez S, Lozano-Marrufo A, Ferré-Eguiluz I, Flores D, Gaitán-Rossi P (2021). Urban poverty and nutrition challenges associated with accessibility to a healthy diet: a global systematic literature review. Int J Equity Health.

[41] Bhowmik B, Siddique T, Majumder A, Mdala I, Hossain IA, Hassan Z (2019). Maternal BMI and nutritional status in early pregnancy and its impact on neonatal outcomes at birth in Bangladesh. BMC Pregnancy Childbirth.

[42] Mehta AR, Panneer S, Ghosh-Jerath S, Racine EF (2017). Factors associated with extended breastfeeding in India. J Hum Lactation Off J Int Lactation Consult Assoc.

[43] Sharma J, Pandey S, Negandhi P (2020). Determinants of suboptimal breastfeeding in Haryana—an analysis of national family health survey-4 data. Indian J Public Health.

[44] Stuebe A (2009). The risks of not breastfeeding for mothers and infants. Rev Obstet Gynecol.

[45] Newman J (1990). Breastfeeding problems associated with the early introduction of bottles and pacifiers. J Hum Lactation Off J Int Lactation Consult Assoc.

